# Musculoskeletal and Head Injuries in a Canadian Summer Camp: A Three-Year Surveillance Study

**DOI:** 10.7759/cureus.24358

**Published:** 2022-04-21

**Authors:** Daniel Freedman, Amalia R Silberman, Daniel Friedberg, Sam Stewart, Katrina F Hurley

**Affiliations:** 1 Department of Family Medicine, Henry Ford Health System, Detroit, USA; 2 Medicine, Queen’s University, Kingston, CAN; 3 Faculty of Medicine, University of Ottawa, Ottawa, CAN; 4 Statistics, Dalhousie University, Halifax, CAN; 5 Department of Emergency Medicine, IWK Health Centre, Halifax, CAN

**Keywords:** treatment, head injury, musculoskeletal injury, residential camp, summer camp

## Abstract

Introduction: Residential summer camps are popular among Canadian families. Campers are exposed to new, unfamiliar environments and engage in activities that may pose increased risk of injury. This study identifies the occurrence and management of musculoskeletal and head injuries at a Canadian residential summer camp.

Methods: This study was a three-year prospective observational cohort study, at a six-week Canadian residential summer camp. There were 1,388 residents, consisting of 51,546 camp days (CD). Injury data were collected by residential summer camp staff and confirmed by onsite medical professionals prior to being recorded in a secure database. Injuries were included if it was a musculoskeletal or head injury that occurred while engaged in a camp activity on or offsite, that necessitated medical attention, and that required removal or restriction from their normal camp routine for a minimum of 4 hours.

Results: There were 154 injuries, resulting in an incidence of 2.99 injuries per 1000 CD. Injuries were reported during scheduled activities (1.46/1000 CD) and free time (1.20/1000 CD). Sports was the most common activity during which injury occurred in all age groups (1.07/1000 CD), where males were injured twice as often as females. 65% of injuries occurred while under staff supervision. The lower extremity was the most affected body part (1.59/1000 CD). Sprains and strains accounted for 1.69 injuries/1000 CD. 83% of injuries were classified as significant and 89% of injuries were treated on-site. Over-the-counter analgesics were provided in 62% of senior camper injuries and 46% of junior camper and staff injuries.

Conclusion: Most injuries in the residential camp setting are mild. Ensuring appropriate non-pharmacologic measures in addition to adequate analgesia may help shorten return to play.

## Introduction

Residential summer camp is a large part of many Canadian families’ lives. Approximately 900,000 Canadian children attend summer camps and the Canadian Camping Association hires over 70,000 staff members each year, most of whom are 17-24 years old [1]. Despite the benefits of camp and physical activity, exposure to new and unfamiliar environments, terrains, and activities may increase the risk of injury for campers and staff.

More than 500,000 children are injured each year in Canada, and re-injury rates increase with age [2]. Children in residential summer camps are less likely to be injured than in youth sports and hospitalization due to injury in this setting is infrequent [3-7]. Although residential summer camp is not more risky than other activities, reducing the occurrence of injuries helps ensure high-quality camp experiences for children and youth [4].

Commonly identified risk factors for injury include inadequate supervision, improper use of protective equipment, and unfamiliar terrain [4,6]. In surveillance studies, sprains and strains have been the leading type of injury in residential summer camps [4-8], most commonly in the lower extremity [4,6,7]. Campers are injured more frequently than staff [4-6], possibly due to anatomic and physiologic differences that make children and adolescents more vulnerable to injury [9]. Diagnosis of concussion has increased in adolescents, attributed to participation in contact sports and increased concussion awareness [10].

The purpose of our study was to describe the occurrence and management of musculoskeletal (MSK) and head injuries in a Canadian residential camp setting.

## Materials and methods

Design

This was a prospective observational cohort study that was conducted at one Canadian residential summer camp. The study took place over three 6-week overnight sessions during the summers of 2017, 2018, and 2019. Campers and residential staff members were interviewed by designated onsite staff, who were provided training prior to the start of the camp session. All data elements were entered from a list of drop-down options into a password-protected, encrypted, electronic offline database. Injury data were confirmed by camp health professionals. The camp director consented to data collection at the camp infirmary and data were de-identified using randomly assigned study identification numbers. Ethics approval was obtained from the Queen’s University Health Sciences & Affiliated Teaching Hospitals Research Ethics Board (file number 6015206).

Setting

While most campers enrolled for a six-week period, some campers stayed for 10- or 21-day periods. Campers remained onsite for the entire duration of camp, with infrequent day trips to nearby areas. Onsite activities included, but were not limited to, swimming, waterskiing, boating, arts and crafts, dancing, and sports. This summer camp was equipped with an infirmary staffed full-time by two senior nursing students and a physician. In the event of an emergency, the nearest emergency department was 31 km away.

Participants

The study cohort consisted of all campers and employed staff who resided at the participating summer camp. Participants were categorized into three groups based on age; “junior campers” (ages 6-11 years), “senior campers” (ages 12-16 years), and “staff” (ages ≥17 years) [6]. Injuries that occurred to kitchen and maintenance staff were not included in our study as they did not reside at camp.

Variables and measures

Injuries were included in our study if they met the following criteria: (1) an MSK or head injury that occurred at any point while engaged in a camp activity on- or offsite that (2) necessitated medical attention, and (3) required removal or restriction from normal camp routine for a minimum of 4 hours [3,5,7]. A head injury was defined as a blow to the head that restricted participant activity; formal concussion testing was not performed. If an individual sustained two separate injuries from the same insult, this was counted as a primary and secondary injury rather than two separate injuries. An injury was deemed “significant” if it removed or restricted activity for 24 or more hours [7]. We excluded abrasions, lacerations, burns, and insect bites or stings, as well as any staff injuries that occurred prior to the arrival of campers.

The primary outcome was the incidence of MSK and head injuries. Secondary outcomes included: timing; activity, and supervision at the time of injury; mechanism of injury; recurrent injury (i.e., whether the same injury had occurred within the previous two years); primary and secondary anatomical locations; duration until return to normal camp routine; where the injury was treated; and treatment(s) provided [6,7].

Statistical and data analysis

We report incidence using “camp days” (CD), defined as one camper or employed residential staff at camp for one day [3-6]. This metric allows for the inclusion of all camp participants, regardless if they stayed for the full six-week session. Descriptive statistics were used to characterize our cohort of camp participants. Cross tabulations with percentages and median values were presented for categorical and continuous data, respectively. Incidence values per 1000 CD were calculated when cumulative days were available for age and sex. P-values were calculated using Fisher’s Exact test where appropriate, and a p-value less than 0.05 was considered significant. All data were analyzed using R Core Team version 4.0.3.

## Results

This study included 1,388 camp residents, consisting of 51,546 CD. The distribution of participants among age categories and their duration of participation is outlined in Table 1. A total of 154 injuries occurred over the three-year period for an incidence of 2.99 injuries per 1000 CD. The incidence of injury was higher in males compared to females across all age groups. Injury occurrences and incidence is reported in Table 2 by age category and sex.

**Table 1:** Cohort demographics and corresponding camp days during the three-year study Jr: junior; Sr: senior; M: male; F: female; CD: camp days; n: number of camp participants

Age group	Total CD (n)	2017 CD (n)	2018 CD (n)	2019 CD (n)
Jr camp M	9829 (319)	3515 (113)	3227 (103)	3087 (103)
Jr camp F	12527 (374)	4225 (130)	4130 (118)	4172 (126)
Jr camp total	22356 (693)	7740 (243)	7357 (221)	7259 (229)
Sr camp M	7056 (168)	2352 (56)	2310 (55)	2394 (57)
Sr camp F	7560 (180)	2310 (55)	2562 (61)	2688 (64)
Sr camp total	14616 (348)	4662 (111)	4872 (116)	5082 (121)
Staff M	6888 (164)	2268 (54)	2394 (57)	2226 (53)
Staff F	7686 (183)	2520 (60)	2688 (64)	2478 (59)
Staff total	14574 (347)	4788 (114)	5082 (121)	4704 (112)
Total	51546 (1388)	17190 (468)	17311 (458)	17045 (462)

**Table 2:** Injury occurrences and incidence by age group and sex Jr: junior; Sr: senior; M: male; F: female; n: number; CD: camp days

Age	Injuries (n)	Injuries/1000 CD
Jr camp M	40	4.07
Jr camp F	34	2.71
Jr camp total	74	3.31
Sr camp M	22	3.12
Sr camp F	17	2.25
Sr camp total	39	2.67
Staff M	26	3.77
Staff F	15	1.95
Staff total	41	2.81
M total	88	3.70
F total	66	2.38
Total	154	2.99

We report injury characteristics in terms of timing, activity, injury type, and outcomes in Table 3. Injuries occurred most often during scheduled activities (1.46 injuries per 1000 CD) followed by free time (1.20 injuries per 1000 CD). Campers and staff were most frequently injured during sports (1.07 injuries per 1000 CD). Basketball was the sport in which injury occurred most often (0.330 injuries per 1000 CD) more than double any other sport. Sprain and/or strain was the most common type of injury (1.69 injuries per 1000 CD). We report the incidence of injury by anatomical location in Table 4. The most frequent anatomical location of injury was the lower extremity (1.59 injuries per 1000 CD). Injuries occurred to a secondary anatomical location 18% of the time. 89% of injuries were treatable onsite; 0.330 injuries per 1000 CD required hospital care where all but one participant was treated and immediately released.

**Table 3:** Activity, injury type, and return to play durations for injuries sustained at residential summer camp Jr: junior; Sr: senior; CD: camp days

	All/1000 CD (%)	Male/1000 CD (%)	Female/1000 CD (%)	Jr camp/1000 CD (%)	Sr camp/1000 CD (%)	Staff/1000 CD (%)
Timing of injury						
Scheduled instructional activity	0.97 (32)	0.94 (30)	1.01 (36)	1.16 (35)	1.10 (41)	0.55 (20)
Scheduled evening program	0.49 (16)	0.50 (16)	0.46 (17)	0.49 (15)	0.41 (15)	0.55 (20)
Free time	1.20 (40)	1.33 (42)	1.05 (38)	1.37 (42)	0.89 (33)	1.24 (44)
Overnight	0.17 (6)	0.18 (6)	0.17 (6)	0.18 (5)	0.07 (3)	0.28 (10)
During meal	0.10 (3)	0.14 (5)	0.04 (2)	0.09 (3)	0.07 (3)	0.14 (5)
Other	0.06 (2)	0.07 (2)	0.04 (2)	0 (0)	0.14 (5)	0.07 (2)
Activity at time of injury						
Sports	1.07 (36)	1.43 (39)	0.76 (32)	1.12 (34)	1.23 (46)	0.82 (29)
Nature	0.35 (12)	0.46 (12)	0.25 (11)	0.63 (19)	0 (0)	0.27 (10)
Incidental/Horseplay	0.78 (26)	0.97 (26)	0.61 (26)	0.98 (26)	0.62 (23)	0.62 (22)
Swim	0.19 (6)	0.25 (7)	0.14 (6)	0.22 (7)	0.14 (5)	0.21 (7)
Boating	0.16 (5)	0.21 (6)	0.11 (5)	0 (0)	0.07 (3)	0.48 (17)
Camp program (in camp)	0.33 (11)	0.21 (6)	0.43 (18)	0.36 (11)	0.34 (13)	0.27 (10)
Camp program (out of camp)	0.02 (1)	0.04 (1)	0 (0)	0 (0)	0.07 (3)	0 (0)
Other	0.10 (3)	0.13 (3)	0.07 (3)	0 (0)	0.21 (8)	0.14 (5)
Nature of injury						
Bruise and/or swelling	0.99 (33)	1.26 (34)	0.76 (32)	1.30 (39)	0.62 (230)	0.89 (32)
Head injury	0.19 (6)	0.29 (8)	0.11 (5)	0.22 (7)	0.14 (5)	0.21 (7)
Fracture and/or dislocation	0.12 (4)	0.17 (5)	0.07 (3)	0.04 (1)	0.27 (10)	0.07 (2)
Sprain and/or strain	1.69 (56)	1.98 (53)	1.44 (61)	1.74 (53)	1.64 (62)	1.65 (59)
Return to play						
4-8 hours	0.14 (5)	0.11 (3)	0.17 (6)	0.18 (5)	0 (0)	0.21 (7)
8-24 hours	0.37 (12)	0.50 (16)	0.21 (8)	0.36 (11)	0.27 (10)	0.48 (17)
24-48 hours	0.41 (14)	0.32 (10)	0.51 (18)	0.45 (14)	0.34 (13)	0.41 (15)
>48 hours	1.75 (58)	1.91 (60)	1.56 (56)	2.19 (66)	1.71 (64)	1.10 (39)
Never returned to normal, remained at camp	0.27 (9)	0.29 (9)	0.25 (9)	0.09 (3)	0.27 (10)	0.55 (20)
Left camp, returned later	0.04 (1)	0.04 (1)	0.04 (2)	0.05 (1)	0 (0)	0.07 (2)
Left camp, did not return	0.02 (1)	0 (0)	0.04 (2)	0 (0)	0.07 (3)	0 (0)
Anatomical location						
Head/Neck	0.39 (13)	0.47 (15)	0.30 (11)	0.40 (12)	0.34 (13)	0.45 (15)
Torso	0.01 (3)	0.11 (3)	0.08 (3)	0.09 (3)	0 (0)	0.28 (7)
Upper extremity	0.91 (31)	1.08 (34)	0.72 (26)	0.81 (24)	0.96 (36)	1.03 (37)
Lower extremity	1.59 (53)	1.51 (48)	1.68 (61)	2.01 (61)	1.37 (51)	1.17 (41)
Treatment location						
Entirely onsite	2.66 (89)	2.77 (88)	2.52 (91)	3.04 (92)	2.26 (85)	2.47 (88)
Offsite and released	0.31 (10)	0.36 (11)	0.25 (9)	0.27 (8)	0.41 (15)	0.28 (10)
Offsite and admitted	0.02 (1)	0.04 (1)	0 (0)	0 (0)	0 (0)	0.07 (2)

**Table 4:** Injury incidence by anatomic location. For data analysis, anatomical locations were classified into four main categories: head & neck, torso, upper extremity, and lower extremity Jr: junior; Sr: senior; CD: camp days

Anatomic Location	All/1000 CD (%)	Male/1000 CD (%)	Female/1000 CD (%)	Jr camp/1000 CD (%)	Sr camp/1000 CD (%)	Staff/1000 CD (%)
Head (including face, scalp, skull)	0.35 (12)	0.40 (12)	0.30 (11)	0.31 (9)	0.34 (13)	0.41 (15)
Neck	0.04 (1)	0.07 (2)	0 (0)	0.09 (3)	0 (0)	0 (0)
Upper back/spine (excluding scapula)	0.04 (1)	0.07 (2)	0 (0)	0 (0)	0 (0)	0.14 (5)
Lower back/spine	0.06 (2)	0.04 (1)	0.08 (3)	0.09 (3)	0 (0)	0.07 (2)
Shoulder (including scapula)	0.25 (8)	0.32 (10)	0.17 (6)	0.22 (7)	0.14 (5)	0.41 (15)
Arm/forearm (including elbow)	0.02 (2)	0 (0)	0.08 (2)	0.05 (1)	0.07 (3)	0 (0)
Wrist	0.27 (9)	0.25 (8)	0.30 (11)	0.22 (7)	0.48 (18)	0.14 (5)
Hand (including fingers, thumb)	0.35 (12)	0.50 (16)	0.17 (6)	0.31 (9)	0.27 (10)	0.48 (17)
Leg (including femur, knee, tibia, fibula)	0.29 (10)	0.40 (12)	0.17 (6)	0.36 (11)	0.14 (5)	0.35 (12)
Ankle	0.93 (31)	0.83 (26)	1.05 (38)	1.25 (38)	0.89 (33)	0.48 (17)
Foot/toes	0.37 (12)	0.29 (9)	0.46 (17)	0.40 (12)	0.34 (13)	0.34 (12)

The incidence of “significant” injuries was 2.05 and 2.99 injuries per 1000 CD in female and male camp participants, respectively. The incidence of “significant” injuries was 2.77, 2.40, and 2.13 injuries per 1000 CD in junior campers, senior campers, and staff, respectively. Forty-seven injury occurrences (31%) were recurrent injuries that were reported to have happened within the preceding two years. Neither age nor sex correlated with recurrent injury. Seventy-one injury occurrences (65%) happened under the supervision of staff. The occurrence of “significant” injury did not correlate with supervision.

Treatments provided by infirmary and hospital staff are listed in Table 5. Ice application (72%) was most often recommended for the treatment of injuries. Over-the-counter (OTC) analgesics were used in campers and staff of all ages to manage MSK and head injuries: junior (46%), senior (62%), and staff (46%).

**Table 5:** Treatment modalities by age category n: total number of treatments that were given to each age group over the three-year period; %: percentage of injuries where the specific treatment was administered; OTC: over the counter

Treatment modality	Jr camp n (%)	Sr camp n (%)	Staff n (%)
None/rest	53 (72)	24 (62)	32 (78)
Ice	53 (72)	26 (67)	24 (59)
Tensor/Brace/Tape	46 (62)	24 (62)	14 (34)
OTC analgesics	34 (46)	24 (62)	19 (46)
Crutches	5 (7)	4 (10)	2 (5)
Cast	1 (1)	3 (8)	0 (0)

## Discussion

We found an incidence of 2.99 injuries per 1000 CD with 0.330 injuries per 1000 CD requiring hospital-based care. This is consistent with other studies [3,6] that have shown a low incidence of severe injury in children and youth at summer camps. Strains and sprains were the most common MSK injury we observed [5,6] and the ankle was the most common anatomic location of injury [4,6]. As in previous studies, sports accounted for the activity with the highest incidence of injury amongst all age groups [4-6] and may relate to a higher level of intense activity during sports or contact between players. A systematic review demonstrated the effectiveness of dynamic warm-up exercises at decreasing the risk of lower limb injuries during sports participation [11] and may point to an effective injury prevention strategy in a camp setting. Further, during daily activities, campers and staff navigate obstacles such as roots and rocks on the camp property. One study noted that campers and staff who experienced trips and falls were often not wearing close-toed shoes with adequate ankle support [4]. We did not collect data about footwear in this study, nor did we collect data on the specific terrain where injuries occurred.

Head injuries accounted for 6% of injuries sustained over the study period; an incidence of 0.194 injuries per 1000 CD, similar to incidences reported by others [4,6,7]. The head injuries that occurred were low impact and without serious morbidity. The benign nature of head injuries in camp settings does not justify concussion baseline testing in residential summer camps. We observed that 31% of injuries were recurrent, where the initial insult took place within two years, exceeding that reported in previous literature [3,5,8]. We did not collect data as to previous injury severity and temporal proximity to the start of the camp session. Future studies may be worthwhile with consideration of the role of active rehabilitation in the camp setting where athletic trainers or physiotherapists are not readily accessible.

MSK and head injuries occurred during scheduled activities and free time [4]. During free time, junior and senior campers experienced injuries at an incidence of 1.37 injuries per 1000 CD and 0.890 injuries per 1000 CD, whereas staff experienced them at 1.24 injuries per 1000 CD. Junior campers engage more readily in horseplay than senior campers (incidence of 0.984 and 0.616 injuries per 1000 CD, respectively), possibly related to senior campers preferring more relaxed activities during their free time. Staff typically use free time for dynamic activities, such as running or recreational sports, which they may not experience during their supervisory duties in the daytime. Almost two-thirds of camper injuries occurred under staff supervision, which has been previously reported [3,7]. We found no relationship between staff supervision and “significant” injuries.

Our study reported both more significant injuries, as well as longer durations for return to camp routines than previously reported [3,7]. This may be partially explained by the nature of the MSK injuries included in our study, contrasted with the inclusion of more benign injuries such as bites, stings, cuts, abrasions, and lacerations included in the aforementioned studies. Our study also demonstrated that “significant” injuries decreased proportionally with age, a finding that may relate to younger campers having increased anxiety while away from their usual home support system. Prolonged recoveries that delay return to camp routines may lower campers’ morale and strain the camper to staff ratio since one staff member is typically assigned to care for the injured camper.

There are no consensus guidelines for return to play in children after MSK injuries. Where certain rehabilitation specialists prefer sport-specific skills, physicians consider pain as an important clinical sign to determine readiness for return to activity [12], pointing to the need for appropriate analgesia. It may also be helpful to compare strength and range of motion to the contralateral extremity as preferred measures for assessing readiness and safety for resumption of normal activity [12].

Our study demonstrated the use of ice as the most common non-pharmacologic treatment for acute MSK injuries. The application of ice to an acute MSK injury such as a sprain or strain and bruise or contusion can be helpful in controlling pain for the first few days [13]. In addition to ice, the other commonly observed treatments for MSK injuries were the use of compression therapy with either a tensor bandage, bracing, or taping. These non-pharmacological treatments follow the rest, ice, compression, and elevation (RICE) principle, a standard treatment protocol for soft-tissue injuries. In the summer camp setting, the RICE principle is often difficult to implement due to the high levels of activity required throughout the day as campers transition from one activity to another.

Pediatric pain management has been studied extensively in emergency department settings, though little is published about how to best manage pain in a residential camp environment where children may experience increased anxiety and distress away from home. Pharmacologic intervention with OTC analgesics such as non-steroidal anti-inflammatory drugs (NSAIDs) or acetaminophen was used in 62% of senior camper injuries, compared to 46% in both junior camper and staff injuries. This may be due to hesitancy to administer medication to younger children while staff may self-medicate rather than seeking treatment at the infirmary [6]. While we viewed NSAIDs and acetaminophen interchangeably in this study, ibuprofen is superior in the pediatric population for mild to moderate pain in acute injury [14-17]. Early administration of ibuprofen may impact return to play in a residential camp setting.

Limitations

Our data were entered using drop-down menus and “trips and falls” was not listed as a mechanism of injury which led to underreporting of this injury type. We did not assess the level of “exposure” to particular activities. For example, we observed more injuries in males than females and did not measure whether males were more likely to engage in higher impact activities that posed a greater risk. Additionally, we considered none and rest as identical treatment modalities, when these are not interchangeable. Finally, our study was conducted under the presumption that all campers desire a prompt return to normal activities. Some campers may have prolonged the recovery process, or exaggerated symptoms to avoid less enjoyable activities.

## Conclusions

Residential summer camps remain a safe environment, where most injuries are mild in nature. Injuries are just as likely to occur in scheduled activities as in free time, and staff supervision did not lead to a reduction in injuries. Junior campers were less often treated with systemic OTC analgesics than their older age counterparts. These findings may help target specific interventions to reduce the impact of MSK and head injuries at Canadian summer camps.
